# Pain and function in patients with chronic low back pain and leg pain after Zhineng Qigong – a quasi-experimental feasibility study

**DOI:** 10.1186/s12891-023-06581-w

**Published:** 2023-06-13

**Authors:** Gabriella Pozarek, Björn Strömqvist, Eva Ekvall Hansson, Gerd Ahlström

**Affiliations:** 1 Department of Health Sciences, Lund University, Lund, Sweden; 2 Departments of Clinical Sciences and Orthopaedics, Lund University, Skåne University Hospital, Lund, Sweden

**Keywords:** Low back pain, Sciatica, Clinical trial, Exercise, Qigong, Rehabilitation, Spinal stenosis

## Abstract

**Background:**

Qigong includes training for body and mind, one method is Zhineng Qigong. Scientific literature on qigong for chronic low back pain (LBP) is sparse. This study aimed to investigate feasibility including evaluation of a Zhineng Qigong intervention for pain and other lumbar spine-related symptoms, disability, and health-related quality of life in patients with chronic LBP and/or leg pain.

**Methods:**

Prospective interventional feasibility study without control group. Fifty-two chronic pain patients (18–75 years) with LBP and/or leg pain (Visual Analogue Scale ≥ 30) were recruited from orthopaedic clinics (spinal stenosis, spondylolisthesis, or segmental pain) and primary healthcare (chronic LBP). Patients from orthopaedic clinics were 1–6 years postoperative after lumbar spine surgery or on lumbar surgery waiting list. Patients received a 12-week training intervention with European Zhineng Qigong. The intervention consisted of face-to-face group activities in non-healthcare setting (4 weekends and 2 evenings per week), and individual Zhineng Qigong training. Main health outcomes were self-reported in a 14-day pain diary, Oswestry Disability Index (ODI), Short Form 36 version 2 (SF-36v2), and EuroQol 5 Dimensions 5 Levels (EQ-5D-5L), once directly before and once directly after the intervention.

**Results:**

Recruitment rate was 11% and retention rate was 58%. Dropouts did not report higher pain (baseline), only 3 dropped out because of lumbar spine-related pain. Adherence was median 78 h group attendance (maximum 94 h) and 14 min daily individual training. Ability to collect outcomes was 100%. Thirty patients completed (mean 15 years symptom duration). Twenty-five had degenerative lumbar disorder, and 17 history of lumbar surgery. Results showed statistically significant (within-group) improvements in pain, ODI, all SF-36v2 scales, and EQ-5D-5L.

**Conclusions:**

Despite low recruitment rate, recruitment was sufficient. A multicentre randomized controlled trial is proposed, with efforts to increase recruitment and retention rate. After this Zhineng Qigong intervention patients with chronic LBP and/or leg pain, also patients with considerable remaining LBP/sciatica after lumbar surgery, had significantly improved in pain and function. Results support involvement of postoperative patients in a future study. The results are promising, and this intervention needs to be further evaluated to provide the most reliable evidence.

**Trial registration:**

NCT04520334. Retrospectively registered 20/08/2020.

**Supplementary Information:**

The online version contains supplementary material available at 10.1186/s12891-023-06581-w.

## Background

Low back pain (LBP) for ≥ 3 months is defined as chronic [1] and is a common health issue associated with disability and decreased health-related quality of life (HRQoL) [2]. Furthermore, LBP frequently has concomitant leg pain [3]. Spine-related pain can be classified into the pain types nociceptive, referred, and neuropathic, where a patient can suffer from a combination of types [4]. Nociceptive pain arises from activation of nociceptors secondary to actual or impending damage to non-neural structures [5] such as from the disc, facet, sacroiliac joint, ligaments, and muscles [4]. Referred pain is perceived at a remote location from the injury, being a segmental component of nociceptive pain. Neuropathic pain is caused by pathology or a lesion in the somatosensory nervous system [5] affecting a spinal nerve root and/or dorsal root ganglion [4]. Radicular pain is a manifestation of neuropathic pain, and radiculopathy is a set of symptoms caused by nerve root pathology [4, 6], also called sciatica [6]. The degenerative lumbar spine disorders (disc herniation, spinal stenosis, spondylolisthesis, and segmental pain) [7] often involve compression of neural structures [8], with symptoms such as LBP, pain in the buttocks/hip/leg/foot, numbness and weakness. Except for compression, other common mechanisms of pain are related to inflammation or microinstability, and a combination of mechanisms may coexist [4].

Numerous studies have evaluated non-surgical interventions for the patient population with chronic LBP and/or leg pain. Several non-pharmacologic therapies, including different types of exercise [9], have shown small to moderate effects for chronic LBP [1]. In a review of clinical guidelines for primary care, it was found that most countries recommend exercise for chronic LBP; however, there was no uniformity in the recommendations regarding a particular type of exercise [9]. For lumbar disc herniation with radiculopathy, there is insufficient evidence on physical therapy/structured exercise programs [10]. A recent systematic review for lumbar spinal stenosis showed moderate-quality evidence from single studies supporting a multimodal approach including manual therapy and exercise, with or without education, being effective [11].

In more severe cases or if non-surgical treatments are unsuccessful, lumbar spine surgery relieves suffering for many patients with degenerative disorders [7]. However, a not insignificant proportion of patients (11–24%), report unchanged or increased pain 1-year postoperatively. Furthermore, neurological abnormalities from impairment of neural structures are often long-standing and may remain [12] or appear after spine surgery, including new pain onset [13]. To our knowledge, training interventions for patients with pain years postoperatively are sparse.

To improve pain and function, there is a need to identify effective non-pharmacologic treatments for radicular LBP [1] and non-surgical treatments for spinal stenosis [11, 14]. It is believed that the demand for the treatment of degenerative spine disorders will further increase [11, 15]. We therefore conclude that there is a need to find non-surgical interventions for improving chronic lumbar spine-related symptoms.

A literature review on qigong research for chronic musculoskeletal pain concluded that qigong may help relieve pain and that more research is needed, as the English language literature is promising but inconclusive [16]. It was also mentioned that effects of qigong are seldom studied on specific pain or musculoskeletal diagnoses. Qigong includes training for both the body and mind [17]. A variety of qigong methods have been used both preventively and against different health conditions. A systematic review found a significant effect of qigong on neck pain or disability in middle-aged or younger adults; however, qigong was not in general more effective than exercise therapy [18]. An earlier systematic review found that evidence for the effectiveness of qigong for pain management was not convincing, mentioning differences between qigong methods and the expertise of teachers among possible important factors [19]. It has been advised that qigong should be learned from a qualified teacher for safety reasons [17]. To our knowledge, the Zhineng Qigong method has not previously been evaluated for lumbar disorders in the English language scientific literature.

It is important that effect studies are well planned and tested and that possible threats are identified and prevented [20]. Therefore, an interventional feasibility study was performed in preparation for a randomized controlled trial (RCT). The study was based on the preliminary hypotheses that the intervention would improve pain and other lumbar spine-related symptoms, disability and HRQoL in patients with chronic LBP and/or leg pain. The aims of the present feasibility study were to 1) investigate recruitment from different patient populations; 2) investigate the retention rate, adherence to the intervention, and the ability to collect outcome measures; 3) evaluate a Zhineng Qigong intervention for pain and other lumbar spine-related symptoms, disability, and HRQoL in patients with chronic LBP and/or leg pain; and 4) perform power calculations for a future RCT.

## Methods

The study design was a quasi-experimental interventional feasibility study without a control group, with one measurement directly before and one directly after the intervention. The Transparent Reporting of Evaluations with Nonrandomized Designs (TREND) statement [21] was used as a guide in the writing of the manuscript, and the intervention was described according to the Template for Intervention Description and Replication (TIDieR) [22].

### Eligibility criteria

A screening tool was developed for the study for eligibility assessment of patients (Table 1). Only a few medical conditions were excluded in the eligibility criteria. Patients were eligible if they had ≥ 3 months of pain with Visual Analogue Scale (VAS, 0–100 mm) score ≥ 30 for the past 2 weeks for LBP and/or leg pain intensity. The patients filled in one VAS for LBP and one for leg pain (due to lumbar disorder) as *the best description of pain for the past 2 weeks*, in accordance with the Swedish spine surgery register (SweSpine) [7]. During a period of 4 months, patients were identified through the following channels (forming the recruitment subgroups):

- SweSpine, patients 1–6 years postoperative (PO) with residual symptoms after lumbar spine surgery (two university hospitals).
- Primary healthcare (PHC) (eight PHC centres).
- The waiting list for lumbar spine surgery (WLS) (one university hospital).

**Table 1:** Eligibility criteria

**Inclusion criteria**
** General**
• Age 18–75 years^a^
• Chronic LBP and/or leg pain (≥ 3 months duration)^a^
• Pain intensity (LBP and/or leg pain “due to lumbar disorder”) ≥ 30 on 0–100 mm VAS, as “the best description of pain for the past 2 weeks”
• Resident in the county of Skåne (southern part of Sweden)
• Comfortable with the Swedish language
• Medical treatments were allowed, but not training of any other qigong, yoga, or meditation during intervention and until 1 month afterwards
**Specific**^a^
***PO patients***
• One surgery, 1–6 years ago, for either spinal stenosis, spondylolisthesis, or segmental pain
• Pain intensity (back and/or leg) ≥ 30 on 0–100 mm VAS in the latest SweSpine follow-up protocol (1, 2, or 5 years postoperative)
***PHC patients***
• Chronic LBP (≥ 3 months duration) with or without leg pain, and having any lumbar spine diagnosis
***WLS patients***
• Planned first surgery for either spinal stenosis, spondylolisthesis, or segmental pain
**Exclusion criteria**
• Lumbar spine or other major surgery planned before, during, or within 1 month after the intervention
• History of serious mental disease, epilepsy, or narcolepsy
• Current abuse of alcohol, medication, or drug
• Pregnancy

*LBP* Low back pain, *PHC* Primary healthcare, *PO* Postoperative after lumbar spine surgery, *SweSpine* Swedish spine surgery register, *VAS* Visual Analogue Scale, *WLS* Waiting list for lumbar spine surgery

^a^The non-self-reported eligibility criteria

### The intervention

The intervention was conducted for 12 weeks with the European Zhineng Qigong school, with which all patients were unfamiliar. Both group activities and individual Zhineng Qigong training were part of the intervention. The group activities (in total 94 h) were held during 4 weekends (12 h each) and 2 evenings per week (2 h each). All group activities were performed face-to-face with the whole group and included lectures, demonstrations and training. An obligatory introductory lecture (2 h) on Zhineng Qigong was given the evening before the first weekend. The training was intensified step-by-step, in accordance with the qigong teacher’s experience. Daily individual training was recommended and was supported by an instructional compact disc (CD S-1, produced by the qigong school). Each patient received this CD.

The training in the intervention consisted of dynamic Zhineng Qigong exercises, with soft patterns of movement which were never forcefully done. The exercises were performed standing up or sitting in a chair if needed. The training included physical movements with concentration and relaxation, with no breath control. The exercises aim to improve homeostasis, thereby achieving a healthier state. During the group activities the patients received detailed instructions about how to perform the exercises and the patients were carefully supervised, with their body postures and movements being corrected if needed. The patients also received instructions not to extend the movements beyond their individual physical ability. One teacher planned and led the intervention and was assisted by 3 additional teachers. All 4 teachers had several years of experience in both training and teaching Zhineng Qigong, having been educated and authorized by the main teacher of the qigong school, a qigong master.

The group activities were performed in a non-healthcare setting in the city of Lund in Skåne, Sweden. The first author (GP) was present to passively observe the progress and reactions of the patients but did not contribute to the intervention.

### Data collection

Identified and interested patients received brief written information about the study. PO patients received it after a phone call by staff or first author (GP), PHC patients from their physician or physiotherapist, and WLS patients after a phone call by staff. Some background data in the screening tool, including lumbar spine diagnoses, was pre-filled and originated from SweSpine or medical records (Table 1, the non-self-reported eligibility criteria). Patients that were interested in receiving more information about the study filled in the self-reported part of the screening tool, including their postal address and telephone number, before it was submitted. The VAS pain intensities (self-reported in the screening tool), for LBP and leg pain respectively, were collected only at study inclusion. Patients who fulfilled the eligibility criteria received an invitation with detailed written information (including the study purpose, the arrangement of the intervention, and the data collection procedure) and a consent form. The eligible patients were also contacted by GP for verbal information by phone, which also allowed them to ask questions. The patients were informed that they would be anonymous in their submitted forms, as these would be signed with a personal code instead of their name, and that GP had no access to the code list.

Health outcomes, all self-reported, were measured with the following: a pain diary (on 0–10 Numerical Rating Scale, NRS) and a general questionnaire (GQ), both developed for this study; the Oswestry Disability Index (ODI) version 2.1a; the Short Form 36 version 2 (SF-36v2); and the EuroQol 5 Dimensions, 5 Levels (EQ-5D-5L). The pain diary and questionnaires were completed once directly before and once directly after the intervention, with no further follow-up.

The patients’ attendance at group activities was registered. Additionally, the patients filled in a training diary with their individual Zhineng Qigong training time each day during the intervention and 2 weeks afterwards (group activities not included).

### Feasibility-related outcomes

The recruitment rate [20] was defined as the percentage of enrolled patients among those who were ‘possibly eligible’. The ‘possibly eligible’ patients were identified according to the non-self-reported eligibility criteria before the self-reported eligibility criteria were filled in (Table 1).

The retention rate [20] was defined as the percentage of patients who completed the study among those who were enrolled.

Adherence to the intervention was evaluated in terms of hours of attendance (at the group activities) and mean daily individual Zhineng Qigong training time in minutes (from the training diary).

The ability to collect outcome measures was defined as the percentage of completed health outcomes at baseline and after the intervention.

### Health outcomes

#### Low back pain and/or leg pain

The pain diary measured the *most usual* pain intensity for LBP and/or leg pain (*due to lumbar disorder*, NRS; 0–10) once daily for 14 days directly before and directly after the intervention. The primary health outcome was the mean pain intensity of each 14-day pain diary. To facilitate the filling in of the pain diary, pain intensity was labelled in the diary completion instructions as none (0), mild (1–3), moderate (4–6) and severe (7–10) [23]. NRS was used in the pain diary, as NRS is commonly used to measure pain and has been validated in pain populations [23]. Furthermore, NRS for pain intensity has been found to be responsive to change in chronic LBP [24].

The GQ measured the number of ‘pain symptoms’ (*n* = 0–4): ‘LBP’, ‘tendency for lumbago’, ‘pain into buttocks/hip/leg/foot’, and ‘pain in both legs’ (Additional file 1). ‘Pain in both legs’ was counted as 2 symptoms, with a maximum score of 2 for leg pain. Patients also scored *how often* they were ‘free from pain’, with 6 time options ranging from ‘almost never’ to ‘completely free’ (Additional file 1). Analgesic intake over the *past 3 months* was assessed at baseline, while after the intervention analgesic intake *since the start of the intervention* was assessed.

#### Other lumbar spine-related symptoms—‘non-pain symptoms’

The GQ measured the number of ‘non-pain symptoms’ (*n* = 0–7): ‘low back weakness/fatigue’, ‘low back sense of instability’, ‘urgency of micturition’, ‘difficulty controlling urine/faeces’, ‘numbness and/or reduced sensory function into buttocks/hip/leg/foot’, ‘weakness/fatigue/reduced function in one leg’, and ‘weakness/fatigue/reduced function in both legs’ (Additional file 1). ‘Weakness/fatigue/reduced function in both legs’ was counted as 2 symptoms, with a maximum score of 2 for leg weakness. Patients also scored *how often* they were ‘free from non-pain symptoms’, with 6 time options ranging from ‘almost never’ to ‘completely free’ (Additional file 1).

#### Disability

The ODI measured spine-related disability *today*, with 10 questions on different aspects (‘pain intensity’, ‘personal care’, ‘lifting’, ‘walking’, ‘sitting’, ‘standing’, ‘sleeping’, ‘sex life’, ‘social life’, and ‘travelling’) [25]. The index was calculated on a scale from 0–100, with lower values being more favourable. The ODI is a valid outcome measure in the management of spinal disorders [25], being valid, reliable, and responsive to change in patients with chronic LBP [24].

#### Health-related qualify of life

The SF-36v2 measured HRQoL (standard *4-week recall*), with 36 generic questions [26, 27]. Eight scales of functional health and well-being (‘physical functioning’, ‘role physical’, ‘bodily pain’, ‘general health’, ‘vitality’, ‘social functioning’, ‘role emotional’, and ‘mental health’) were transformed into values using the original 0–100 scoring, with higher scores being more favourable. The scale scores were aggregated into a physical component summary (PCS) and a mental component summary (MCS), both with a mean score of 50 based on the 2009 U.S. general population sample. QualityMetric Health Outcomes™ Scoring Software version 4.5 was used (OptumInsight Life Sciences, Inc.). The Short Form 36 has been shown to be a valid, reliable, and responsive outcome measure in patients with chronic LBP [24].

The EQ-5D-5L measured generic HRQoL *today* in 5 dimensions (‘mobility’, ‘self-care’, ‘usual activities’, ‘pain/discomfort’, and ‘anxiety/depression’), each with 5 severity levels [28, 29]. The index, where 1.00 is the most favourable, was calculated with EQ-5D-5L index value calculator version 1.1 (Denmark value set), with a crosswalk between EQ-5D-5L and EQ-5D-3L [30]. The EQ-5D-5L also includes a vertical scale, the EuroQol Visual Analogue Scale (EQ VAS), where overall health is scored 0–100, with higher scores being more favourable [28]. For patients with LBP, EuroQol 5 Dimensions has shown good validity and responsiveness [31].

The GQ measured 7 additional aspects of HRQoL in *the past week* (NRS; 0–10): ‘concentration’, ‘stressed out’, ‘sleep’, ‘energy level’, ‘sad or depressed’, ‘irritable’, and ‘tense or anxious’ (Additional file 1).

### Statistics

Because of the small sample size and discrete nature of several outcomes, the descriptive statistics are presented as medians and quartiles. For within-person changes in ordinal or continuous data, the Wilcoxon signed ranks test was used, and for dichotomous data, the McNemar test was used. Since confidence intervals based on medians could be inappropriate, especially since the sample size was small, means of changes are presented with 95% confidence intervals calculated using the paired samples t-test. All reported *p *values are, however, from the non-parametric tests.

A priori power calculations were not performed due to lack of information on this intervention for this population. The power calculations for a future RCT were based on the results from the present study using G*Power version 3.1.9.4 with a statistical power of 0.80 and alpha of 0.05 (independent samples t-test; two-tailed).

In the between-group analyses of ordinal or continuous data, the Mann–Whitney U test was used, and for nominal data, Fisher’s exact test was used.

Analyses were performed using IBM SPSS Statistics version 22. All *p* values were exact, two-tailed, with < 0.05 regarded as statistically significant.

## Results

### Participant flow

**Fig. 1:**
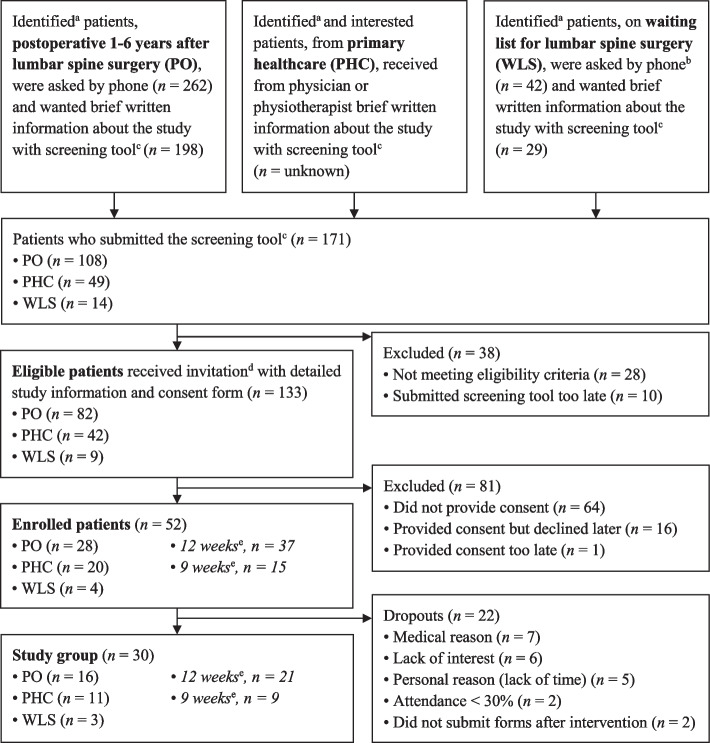
Flowchart of the recruitment process and formation of the study group ^a^‘Possibly eligible’ patients were identified according to the non-self-reported eligibility criteria (Table 1) ^b^Patients were informed during the initial phone call that their participation would not affect their planned surgery date ^c^Screening tool with self-reported Visual Analogue Scale for low back pain and leg pain intensity respectively, for the past 2 weeks, and questions (yes/no) on additional eligibility criteria (Table 1) ^d^Eligible patients were also provided with verbal information by phone ^e^The original intervention was 12 weeks long, and a group of patients joined after 3 weeks

As enrolment initially progressed slowly, the study also enrolled patients for a 3 weeks shorter intervention. A separate introductory lecture and starting weekend was arranged for the 9-week group, after which these patients joined those who had already started the original 12-week intervention. Thirty-seven patients were enrolled for 12 weeks and 15 for 9 weeks (Fig. 1), with group activities held for 94 h and 82 h, respectively. Subgroup analyses were performed for patients who participated 12 or 9 weeks. As the health outcome results for these subgroups were not significantly different from each other, the results are presented for the whole study group without considering the length of the intervention.

In addition, patients with VAS pain intensity score of 25–30 became eligible if their pain situations were qualitatively assessed to be severe. This assessment was performed through a structured telephone interview by the first author (GP) or was self-reported in the screening tool, resulting in enrolment of 4 patients and 1 patient, respectively.

In total, 52 of the 171 patients who submitted the screening tool were enrolled (Fig. 1).

### Feasibility

#### Recruitment

The recruitment rate was approximately 11% based on the estimation that 190 PHC patients were ‘possibly eligible’, with the assumption that the recruitment rate of PHC patients was proportionate to that of the patients from orthopaedic clinics (Fig. 1). It remains unknown how many PHC patients were informed about the study.

#### Retention

The retention rate was 58%, with the reasons for dropout shown in Fig. 1. The dropouts did not report higher pain intensity than the study group at study inclusion or at baseline. Of the 7 individuals who dropped out for ‘medical reason’, 1 was most likely related to chronic medical or psychological disorders and 3 were related to such disorders. Three patients (PO patients), reporting severe pain at study inclusion, communicated that they dropped out because of lumbar spine-related pain. Compared to the study group, the dropouts reported (at baseline) a lower frequency of weakness/fatigue/reduced function in the leg(s) (*p* = 0.021, continuous data).

#### Adherence

For the whole study group, the median group activity attendance was 78 h (IQR 54–80). For the patients who could attend 94 h (12 weeks), the median was 76 h (IQR 54–84), while for those who could attend 82 h (9 weeks), the median was 79 h (IQR 50–80).

The training diaries showed a median of 14 min (IQR 10–18) of daily individual qigong training for the whole study group. The patients who could attend 94 h, had median 14 min (IQR 11–18), while those who could attend 82 h, had median 11 min (IQR 3–20).

#### Ability to collect outcome measures

At baseline, 99.6% of health outcomes were collected (pain diary 100%, GQ 100%, ODI 100%, SF-36v2 99.5%, EQ-5D-5L Index 100%, and EQ VAS 98.1%) (*n* = 52). After the intervention, 100% of health outcomes were collected (*n* = 30). This resulted in an overall ability to collect outcome measures of 99.7%.

### Background data of the study group

The background data of the study group (*n* = 30), having a mean age of 57 (SD 11), is shown in Table 2. The mean VAS pain intensity scores at study inclusion were 55 for LBP (SD 22) and 50 for leg pain (SD 29). The mean symptom duration for lumbar spine/leg(s) was 15 years (SD 14). Seventeen patients had a history of lumbar surgery (16 PO patients and 1 WLS patient). Two PO patients had undergone surgery twice. Twenty-five patients were diagnosed with a degenerative lumbar spine disorder, and 5 (PHC patients) had non-specific LBP with radiation. Twenty-nine patients reported at least one additional disease or disorder, such as rheumatic disease, neurological disease, osteoarthritis, fibromyalgia, osteoporosis, dizziness, neck pain, shoulder pain, tension type headache, migraine, and tinnitus.

**Table 2:** Background data of the study group (*n* = 30)

Characteristics	Mean (SD) or *n* (%)
Age (years)	57.4 (11.4)
Gender, female	21 (70%)
Working level	
Full-time	5 (17%)
Part-time	9 (30%)
Not working^a^	16 (53%)
University education	13 (43%)
Married or living with partner	22 (73%)
Current smoker	4 (13%)
Recruitment subgroup	
PO patients	16 (53%)
Surgery 1–2 years ago	3
Surgery 2–3 years ago	6
Surgery 3–4 years ago	5
Surgery 4–6 years ago	2
PHC patients	11 (37%)
WLS patients	3 (10%)
Patients with pain at study inclusion	
LBP	29 (97%)
Leg pain	29 (97%)
VAS pain intensity at study inclusion	
LBP (0–100)	54.7 (22.4)
Leg pain (0–100)	50.2 (29.5)
Analgesic intake past 3 months	26 (87%)
Duration of lumbar/leg symptoms (years)	14.9 (14.4)
Primary lumbar spine diagnosis	
Spinal stenosis	12 (40%)
Central	6
Lateral	6
Spondylolysis-olisthesis	5 (17%)
Segmental pain	3 (10%)
Disc herniation	5 (17%)
Lumbago-sciatica, lumbago or back pain	5 (17%)

*LBP* Low back pain, *PHC* Primary healthcare, *PO* Postoperative after lumbar spine surgery, *SD* Standard deviation, *VAS* Visual Analogue Scale, *WLS* Waiting list for lumbar spine surgery

^a^Seven patients were aged 66–75 years

### Health outcomes of the study group

#### Decreased low back pain and/or leg pain

**Fig. 2:**
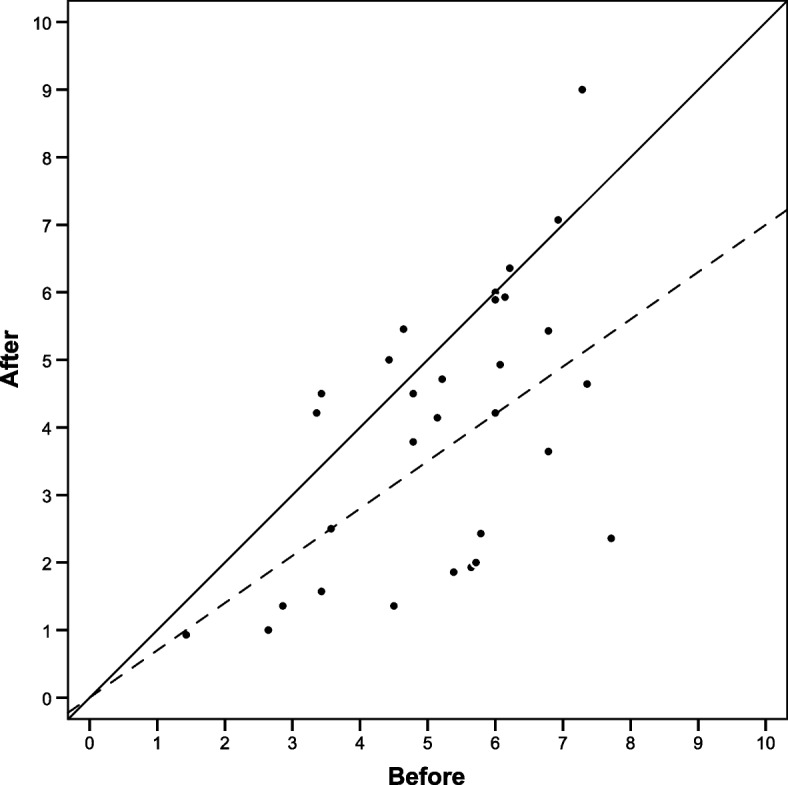
Pain intensity. The mean pain intensity of each 14-day pain diary (lumbar spine-related, NRS 0–10), with improvement for the study group (*p* < 0.001, *n* = 30). The median before the intervention was 5.5 (IQR 4.2–6.2) and after intervention it was 4.2 (IQR 2.0–5.4). The mean change with the 95% confidence interval was -1.24 (-1.88 to -0.60). Patients below the solid line improved, and those below the dashed line improved more than 30%

**Fig. 3:**
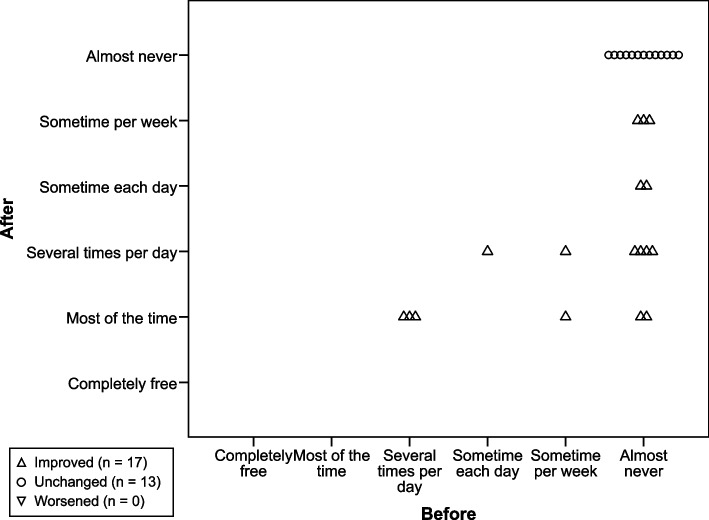
Time ‘free from pain’. How often patients were ‘free from pain’ (low back pain and/or leg pain), with improvement for the study group (*p* < 0.0001, *n* = 30)

Pain intensity decreased (*p* < 0.001, Fig. 2), and time ‘free from pain’ increased (*p* < 0.0001, Fig. 3). For 13 patients (43%) pain intensity decreased ≥ 30%. The pain diaries showed that no patient reported any days without pain at baseline; however, after the intervention, 5 patients had a total of 23 pain-free days.

Four patients completely stopped taking analgesics, and 12 reduced their consumption, of whom 10 reduced their intake *considerably*. Ten patients had unchanged consumption. Four patients consumed no analgesics neither over the 3 months before, nor during the intervention. Nobody increased their intake.

#### Relief of ‘non-pain symptoms’

**Fig. 4:**
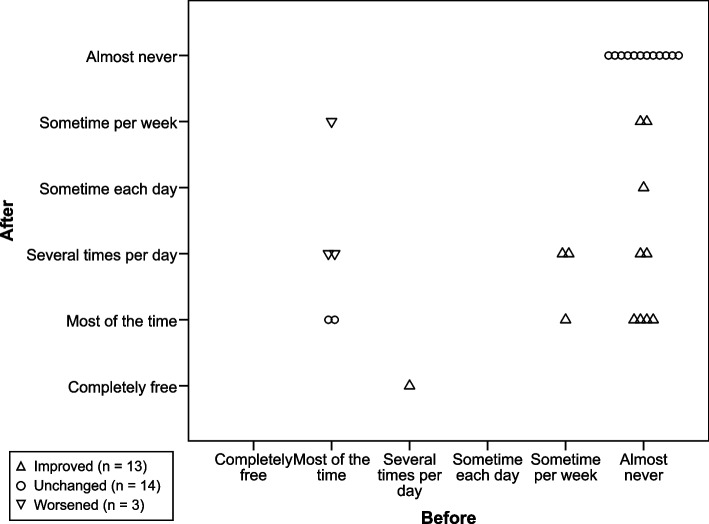
Time ‘free from non-pain symptoms’. How often patients were ‘free from non-pain symptoms’ (lumbar spine-related), with improvement for the study group (*p* = 0.005, *n* = 30)

The number of ‘non-pain symptoms’ decreased (*p* = 0.004, Table 3), and the time ‘free from non-pain symptoms’ increased (*p* = 0.005, Fig. 4). ‘Urgency of micturition’ ceased for 6 of the 10 patients reporting this symptom at baseline (*p* = 0.031).

**Table 3:** Lumbar spine-related symptoms, disability, and HRQoL in the study group (*n* = 30)

Domain	Instrument	Variable	Before intervention Median (IQR)	After intervention Median (IQR)	Change Mean (95% CI)	*p* value
Lumbar spine- related symptoms^a^	*GQ*^(^^−^^)^	Pain symptoms	2.0 (1.0–3.0)	2.0 (1.0–3.0)	0.1 (-0.2, 0.3)	0.795
		Non-pain symptoms	3.0 (2.0–4.0)	2.0 (0.0–3.0)	-0.8 (-1.4, -0.3)	**0.004**
Disability	*ODI*^(^^−^^)^	Index	35.0 (25.5–44.0)	26.0 (17.5–42.0)	-8.2 (-11.8, -4.7)	** < 0.0001**
HRQoL	*SF-36v2*^(+)^	Physical functioning	50.0 (38.8–65.0)	70.0 (48.8–80.0)	13.3 (8.0, 18.7)	** < 0.0001**
		Role physical^*^	43.8 (25.0–68.8)	62.5 (42.2–93.8)	18.0 (7.6, 28.3)	**0.001**
		Bodily pain	36.5 (24.2–43.5)	51.5 (41.0–64.5)	18.0 (12.3, 23.7)	** < 0.000001**
		General health	47.5 (26.5–75.5)	63.5 (33.8–83.2)	8.7 (2.4, 15.1)	**0.002**
		Vitality	31.2 (17.2–51.6)	56.2 (34.4–70.3)	17.7 (10.1, 25.3)	** < 0.0001**
		Social functioning	62.5 (50.0–78.1)	75.0 (59.4–100.0)	13.3 (5.2, 21.5)	**0.002**
		Role emotional^*^	75.0 (50.0–91.7)	91.7 (68.8–100.0)	11.2 (2.0, 20.5)	**0.017**
		Mental health	60.0 (53.8–70.0)	80.0 (63.8–90.0)	12.0 (5.9, 18.1)	** < 0.001**
		PCS^*^	37.6 (31.0–43.6)	40.9 (36.3–50.1)	5.7 (2.9, 8.6)	** < 0.001**
		MCS^*^	47.3 (36.9–50.6)	54.2 (45.4–58.2)	5.7 (2.2, 9.2)	**0.004**
	*EQ-5D-5L*^(+)^	Index	0.62 (0.48–0.72)	0.71 (0.64–0.79)	0.09 (0.04, 0.14)	**0.003**
		EQ VAS^*^	50.0 (39.0–70.0)	72.5 (53.8–80.0)	13.3 (6.8, 19.8)	** < 0.001**
	*GQ* (*NRS*; *0–10*)	Concentration^(+)^	7.0 (5.0–8.0)	8.0 (5.8–9.0)	0.6 (0.0, 1.2)	0.059
		Stressed out^(^^−^^)^	5.0 (2.0–6.2)	4.0 (2.0–7.2)	0.1 (-0.8, 1.0)	0.769
		Sleep^(+)^	5.0 (3.0–7.0)	7.5 (5.0–8.2)	1.8 (1.0, 2.7)	** < 0.001**
		Energy level^(+)^	5.0 (3.0–6.0)	7.0 (5.0–8.0)	1.8 (1.1, 2.4)	** < 0.0001**
		Sad or depressed^(^^−^^)^	3.0 (1.8–7.0)	2.0 (0.0–4.2)	-0.7 (-1.8, 0.3)	0.172
		Irritable^(^^−^^)^	3.0 (1.8–7.0)	3.5 (1.0–6.0)	-0.4 (-1.4, 0.7)	0.184
		Tense or anxious^(^^−^^)^	3.5 (2.0–6.0)	3.5 (1.0–5.2)	-0.4 (-1.7, 0.8)	0.395

*CI* Confidence interval, *EQ-5D-5L* EuroQol 5 Dimensions 5 Levels, *EQ VAS* EuroQol Visual Analogue Scale, *GQ* General questionnaire, *HRQoL* Health-related quality of life, *IQR* Interquartile range (Q1–Q3), *MCS* Mental component summary, *NRS* Numerical Rating Scale, *ODI* Oswestry Disability Index, *PCS* Physical component summary, *SF-36v2* Short Form 36 version 2

^(^^−^^)^Low score favourable

^(+)^High score favourable

^*^One missing value before the intervention. Bold numbers indicate statistically significant differences

^a^Presented as the number of ‘pain symptoms’ (*n* = 0–4) and the number of ‘non-pain symptoms’ (*n* = 0–7)

#### Relieved disability and improved health-related qualify of life

After the intervention, improvements were found in ODI and all 8 SF-36v2 scales, as well as for PCS, MCS, EQ-5D-5L index, EQ VAS, ‘sleep’, and ‘energy level’ (all, *p* ≤ 0.017, Table 3). Three patients (10%) had an ODI score ≤ 20 at baseline; however, 10 (33%) had ODI scores ≤ 20 after the intervention.

### The majority of postoperative patients had decreased pain intensity

Among the PO patients (*n* = 16) having a mean age of 63 (SD 8), 12 had spinal stenosis and 4 had spondylolysis-olisthesis. At study inclusion the patients with spinal stenosis were distinguished by severe leg pain intensity, mean VAS score 64 (SD 25).

For the PO patients, the mean change in pain intensity with 95% confidence interval was -1.88 (-2.78 to -0.98), *p* < 0.001. Fourteen (88%) had decreased pain intensity, whereof 7 (44%) had decreased pain intensity of ≥ 30%. At baseline, 14 patients (88%) reported being *almost never* ‘free from pain’, and 12 (75%) reported being *almost never* ‘free from non-pain symptoms’; both decreased to 7 (44%) after the intervention. All statistically significant improvements for the study group were also significant for the PO patients (all, *p* ≤ 0.049), except for time ‘free from non-pain symptoms’ (*p* = 0.055), ‘urgency of micturition’ (*p* = 0.250), and ‘role emotional’ (*p* = 0.145). Furthermore, the PO patients had made improvements in ‘concentration’ (*p* = 0.037) and ‘irritable’ (*p* = 0.045).

### Patients with VAS pain intensity < 30

Two of the 5 patients with VAS scores of 25–30 completed the study. At baseline, they had pain intensity scores of 5.7 (PO patient) and 3.4 (PHC patient). All statistically significant improvements for the study group were also significant if these 2 completers were excluded (all, *p* ≤ 0.031). In this subgroup (*n* = 28), ‘concentration’ also improved (*p* = 0.047).

### Power calculations

In the power calculations for a future RCT, we assumed that the patients would have similar baseline levels and SDs for pain intensity (primary health outcome) as in the present study. Furthermore, we assumed that the control group would also improve. Two different scenarios were used for the power calculations, with a statistical power of 0.80 and alpha of 0.05:

- In the first scenario, we approximated the mean difference in pain intensity between groups (study group and control group) to 1 and the SD to 2, resulting in 64 patients per group.
  For postoperative patients, we approximated the mean difference in pain intensity to 1.5 and the SD to 1.8, resulting in 24 patients per group.
- In the second scenario, we assumed that the pain intensity in a waiting list control group would decrease by 13%, as was observed in a somewhat similar study [32]. Based on the proportional improvement in that study, we estimated the mean difference in pain intensity to 0.57 between groups and the SD to 2, resulting in 195 patients per group.
  For postoperative patients, we estimated the mean difference in pain intensity to 1.13 and the SD to 1.8, resulting in 41 patients per group.

There will still be dropouts in an RCT, and given the same dropout frequency as in the present study (Fig. 1), additionally 73% patients would be needed, and 75% for postoperative patients.

## Discussion

The present study was conducted to study a Zhineng Qigong intervention in terms of feasibility and subjective health outcomes in patients with chronic LBP and/or leg pain from lumbar disorder. Patients were recruited from orthopaedic clinics and primary healthcare, where postoperative patients were found to be the most feasible for involvement in a future RCT. Qigong is still rather unknown and there is limited evidence of its effects on pain, which might explain the difficulties recruiting and a retention rate lower than expected. Interestingly, all validated health outcomes were significantly improved (within-group) after this intervention.

Three RCTs for chronic LBP were included in the literature review on qigong for chronic musculoskeletal pain [16], for adults [33], older adults [32], and office workers [34]. After 12-week interventions, the respondents reported non-significant difference in pain intensity compared to exercise therapy [33] and waiting list control [32], respectively. The third RCT [34] showed significantly decreased pain intensity compared to waiting list control after a 6-week intervention. As the present study was an interventional feasibility study without control group, in contrast to the 3 RCTs, the results for health outcomes are not directly comparable.

The retention rate was higher in the RCTs which were performed in Germany and Thailand. Compared to this study, some spine-related and additional medical conditions were to varying degrees excluded and there were restrictions in analgesic intake. This may indicate a more severe pain situation for the enrolled patients in the present study who also had leg pain, which probably contributed to difficulties participating. Furthermore, qigong may be more familiar to people in these countries [35, 36] compared to Sweden [37], and in the RCTs fewer sessions were organized, which is likely to have positively affected the retention rate.

A clinical reasoning mentioned in the literature review, is that qigong through its postures and gentle movements performed with concentration and relaxation may reduce both physical and psychological symptoms, that may relieve pain and increase wellbeing [16]. Several hypothetical outcomes of qigong training were mentioned that potentially could impact pain positively, such as improvements in blood flow, functional ability, muscle endurance, strength and tone, reduced stiffness of joints as well as improvements in mental function, mood and sleep quality. Possible underlying mechanisms for pain associated qigong outcomes need to be further investigated. Older adults have as a group been considered more difficult to treat. Among explanations may be a higher prevalence of both co-morbidities as well as specific causes in the spine [32], and nearly all persons of higher ages have spinal degeneration [15]. In the present study there was a wide range of ages, with a relatively high mean age particularly for the PO patients. However, we have not observed any negative influence of age on the health outcomes.

In qigong interventions for chronic musculoskeletal pain several factors may possibly influence the results, including pain history, medical conditions, and qigong teacher characteristics and expertise [16]. More research is recommended in these areas, such as investigating the quality, duration and frequency of qigong training needed to achieve possible positive pain effects in different samples. Therefore, it may be difficult to generalize results from one qigong intervention to another.

### Feasibility

#### Recruitment

The recruitment rate was lower than expected, highlighting both the need to increase the quantity of ‘possibly eligible’ patients and to increase interest during recruitment in a future RCT. During the provision of the verbal information to eligible patients, several were interested but found the intervention too time consuming. Probably, the recruitment rate would increase with fewer scheduled hours.

Concerning the recruitment of PHC patients, we believe that the number of patients who submitted the screening tool was low (*n* = 49), as chronic LBP is a common condition and 8 PHC centres were involved during 4 months. One reason for the low submission may be that healthcare professionals may not have found the time to engage in the study or did not recommend the intervention because of a lack of evidence for qigong.

In future studies, a longer recruitment period may give access to a larger quantity of ‘possibly eligible’ patients. However, patients who have a planned surgery usually cannot wait long for this kind of intervention to start. Furthermore, a longer recruitment period may increase early dropouts before the intervention starts, as motivation to participate might decline over time or changes in living conditions may occur. Additionally, it may be more difficult to involve healthcare professionals for a longer period. A multicentre RCT can potentially be more efficient than a single-centre study and might possibly be performed sequentially with one centre at a time.

Written information with health outcome results from the present study could increase interest among both patients and healthcare professionals. Furthermore, an educational session aiming to increase patients’ awareness of the studied health problem could be arranged, which has been shown to increase the recruitment rate [38].

#### Retention

Several patients communicated that they already had pain relief early during the intervention, while others experienced pain during the training. In a qualitative study involving individuals with chronic non-specific LBP, it has been pointed out that since exercise often does not give immediate pain reduction, it may be hard to recognize its benefits [39]. Also, the participants found it difficult to maintain their motivation to continue with self-management strategies, leading to poor adherence to advice and exercise. The importance of the participants’ willingness to accept activity despite pain was mentioned. In the present study, we cannot rule out that several patients may have avoided the intervention because of their pain, even though only 3 patients said they dropped out because of lumbar spine-related pain.

It is worth noting that as many as 16 patients first provided consent but declined to participate before the baseline outcome measures were collected. The intervention required time and effort, and several enrolled patients had other diseases, such as rheumatic disease, neurological disease, fibromyalgia, and osteoarthritis, in addition to chronic pain from lumbar disorder. The dropouts due to ‘medical reason’ should be viewed in light of few medical exclusion criteria. Almost no co-morbidities were excluded, based on experience that persons with many different health conditions experienced improvements by this intervention. The few medical exclusion criteria made it more likely that the enrolled patients were rather representative of typical patients with chronic LBP and/or leg pain.

It has been mentioned that qigong practice may not suit everyone, as it requires frequent training [16]. One suggestion for future research studies is to arrange a lecture for eligible patients where they get an opportunity to try the training before enrolment. Furthermore, as the patients suffer from chronic pain, the intervention may start with several consecutive days with overnight stays which might help with compliance. No significant differences between patients participating 12 or 9 weeks were shown, indicating that an intervention could be somewhat shorter which might also increase the retention rate.

#### Adherence

Adherence both for group activities and individual training was considered adequate. Concerning attendance, it should be noted that the patients had chronic pain and several of them spent a substantial time travelling to the group activities. Regarding the training diary (only measuring individual training), it was also filled in for days with group activities and thus did not reflect the total daily training time.

The arrangement with regular weekly group activities, together with the instructional CD, made it easier to perform the individual training. The training diary was also a reminder to practise and it was probably motivating.

#### Ability to collect outcome measures

The collection of outcome measures showed a high level of data completion. Several questionnaires, as well as the pain diary, were collected to cover the preliminary hypotheses. While giving the verbal information before enrolment, patients were informed about the importance of completing all the forms.

### Evaluation of the intervention

The result showed statistically significantly reduced chronic LBP and/or leg pain (within-group) after this intervention. For pain intensity, which was the primary health outcome, 43% of patients achieved the minimal clinically important change as suggested to be 30% improvement [40].

Patients reported experiencing symptoms for 15 years in mean, and most (83%) were diagnosed with a degenerative lumbar spine disorder. At baseline, 80% reported that they *almost never* had time ‘free from pain’, which significantly improved after the intervention, as well as ODI and most HRQoL outcomes. The finding that all SF-36v2 scales were significantly improved, supports [26] that Zhineng Qigong works on both physical and mental aspects including an increase in vitality. After the intervention, one-third of the patients reported ODI scores ≤ 20, which has been proposed as minimal disability [25, 41]. Pain intensity was found to decrease in conjunction with increased time ‘free from pain’ and a reduction in analgesic intake, strengthening the indication of an improved pain situation. We find these results valuable for future research on this intervention, which might later become useful for training motivated patients with chronic lumbar disorders.

Several patients probably had symptoms caused by disruption in neural structures, as 73% had a structural diagnosis (disc herniation, spinal stenosis, or spondylolysis-olisthesis) and 57% previously underwent lumbar surgery. One such symptom is ‘urgency of micturition’, which was significantly reduced in number. Also the PO patient subgroup reported significant improvements in pain intensity, time ‘free from pain’, and the number of ‘non-pain symptoms’. These improvements are interesting, as the degenerative process normally continues even if surgery relieved mechanical obstruction [8]. However, this study was conducted to evaluate feasibility including health results, while the mechanisms behind why patients could improve or not, were outside the aim of this study.

### Power calculations

Sample size calculations based on results from feasibility studies are uncertain and should be used cautiously because of limited sample sizes [20]. Since our power calculation scenarios were based on assumptions of change in pain intensity after 3 months in fictive control groups, the estimated sample sizes are even more uncertain. According to the sample size estimations, both power calculation scenarios were shown to be feasible for the PO patient subgroup. However, it is important to increase the retention rate in a future RCT. It should be noted that the control group from the study [32] used for assumptions in the second scenario may not be representative of typical patients after lumbar surgery included in pain trials.

### Strengths and limitations

In addition to recruiting PHC patients, it was decided to target PO and WLS patients with spinal stenosis, spondylolisthesis, or segmental pain. These diagnoses were chosen as they are associated with somewhat lower surgery satisfaction rates. One year postoperatively, unchanged or increased pain has been reported in 15–24% (back) and 13–23% (leg) [7]. It is a novelty to evaluate a training intervention where many patients have considerable residual symptoms years postoperatively. This subgroup’s significant improvements in pain, ODI, 7 of the 8 SF-36v2 scales, and EQ-5D-5L constitute a strength of this study.

The present study was initiated in response to feedback from persons with neuropathic pain and chronic diseases who experienced symptomatic improvements and restoration of functions when training with the qigong school engaged in this study. Qigong’s potential effectiveness for structural diagnoses is still a rather new concept, with sparse evidence in the scientific literature. The results of the present study were in line with the preliminary hypotheses, with all validated outcome measures significantly improved in this rather small sample, strengthening the rationale for a future RCT.

The study limitations are lack of control group and long-term follow-up, and relatively small sample size. The study did not control for unspecific effects such as Hawthorne, placebo, and regression towards the mean. Also, sample heterogeneity is a limitation. However, our largest recruitment subgroup (PO patients) is generally expected to have more difficulty improving through training, but showed similar improvements to the whole study group. Additionally, as the GQ was developed for this study and is not validated, we suggest that its findings be interpreted with caution as well as the power calculations.

## Conclusions

Despite a somewhat low recruitment rate, the expected number of patients was enrolled during the short recruitment period, making recruitment sufficient. A multicentre RCT with a longer recruitment time and possibly somewhat shorter intervention than in this feasibility study are proposed, with efforts to increase the recruitment and retention rate. After this Zhineng Qigong intervention patients with chronic LBP and/or leg pain, also patients with considerable remaining LBP/sciatica after lumbar spine surgery, had significantly improved in self-reported pain, function, and HRQoL. The health results for the PO patients support involvement of postoperative patients in a future study. The results are promising, and this intervention needs to be further evaluated to provide the most reliable evidence through larger sample size, control group, adequate blinding, and long-term follow-up.

## Supplementary Information

**Additional file 1.** Questions in the general questionnaire for lumbar spine‑related symptoms and HRQoL.

## Data Availability

The datasets used and/or analyzed during the current study are available from the corresponding author on reasonable request.

## Abbreviations

CD: Compact disc
EQ-5D-3L: EuroQol 5 Dimensions, 3 Levels
EQ-5D-5L: EuroQol 5 Dimensions, 5 Levels
EQ VAS: EuroQol Visual Analogue Scale
GQ: General questionnaire
HRQoL: Health-related quality of life
IQR: Interquartile range, presented with quartile 1 and quartile 3 (Q1–Q3)
LBP: Low back pain
MCS: Mental component summary
NRS: Numerical Rating Scale
ODI: Oswestry Disability Index
PCS: Physical component summary
PHC: Primary healthcare
PO: Postoperative after lumbar spine surgery
RCT: Randomized controlled trial
SD: Standard deviation
SF-36v2: Short Form 36 version 2
SweSpine: Swedish spine surgery register
TIDieR: Template for Intervention Description and Replication
TREND: Transparent Reporting of Evaluations with Nonrandomized Designs
VAS: Visual Analogue Scale
WLS: Waiting list for lumbar spine surgery
