# Supplementary material for: Pain and function in patients with chronic low back pain and leg pain after Zhineng Qigong – a quasi-experimental feasibility study
Source: BMC Musculoskelet Disord. 2023 Jun 13;24:480. doi: 10.1186/s12891-023-06581-w (PMC10262421; doi:10.1186/s12891-023-06581-w)
Supplement: Supplementary file 1 — Additional file 1. Questions in the general questionnaire for lumbar spine‑related symptoms and HRQoL. [file 12891_2023_6581_MOESM1_ESM.docx]

**Additional file 1** Questions in the general questionnaire for lumbar spine‑related symptoms and HRQoL

| **Domains** | **Questions** | **Answers alternatives** |
| --- | --- | --- |
| **Pain symptoms** | Which lumbar spine‑related pain symptoms do you have? | - Low back pain - Tendency for lumbago - Pain into buttocks/hip/leg/foot *** - Pain in both legs ***   ** If pain in both legs: maximum 2 symptoms* |
|  | If you think specifically of pain in lumbar spine or leg(s) (due to lumbar problem), how often are you free from these types of pain? | - Almost never - Sometime per week - Sometime each day - Several times per day - Most of the time - Completely free |
| **Non‑pain symptoms** | Which lumbar spine‑related symptoms, except for pain, do you have? | - Low back weakness/fatigue - Sense of instability in the low back - Need to visit the toilet more often than normal because of urgency of micturition - Difficulty controlling urine or faeces - Numbness and/or reduced sensory function into buttocks/hip/leg/foot - Weakness/fatigue/reduced function in one leg ** - Weakness/fatigue/reduced function in both legs **   *** If weakness/fatigue/reduced function in both legs: maximum 2 symptoms* |
|  | Except for pain, how often are you free from your other lumbar spine‑related symptoms above? | - Almost never - Sometime per week - Sometime each day - Several times per day - Most of the time - Completely free |
| **HRQoL** | How would you rate your concentration ability in the past week? | NRS; 0–10:  0 = Very poor; 10 = Very good |
|  | How stressed out have you been in the past week? | 0 = Not at all; 10 = Very stressed out |
|  | How would you rate your sleep in the past week? | 0 = Very poor; 10 = Very good |
|  | How would you rate your energy level in the past week? | 0 = Very low; 10 = Very high |
|  | Have you felt sad or depressed in the past week? | 0 = Not at all; 10 = Very sad or depressed |
|  | Have you felt irritable in the past week? | 0 = Not at all; 10 = Very irritable |
|  | Have you felt tense or anxious in the past week? | 0 = Not at all; 10 = Very tense or anxious |

*HRQoL* Health‑related quality of life, *NRS* Numerical Rating Scale
